# Fluid–Structure Interaction Simulations of the Initiation Process of Cerebral Aneurysms

**DOI:** 10.3390/brainsci14100977

**Published:** 2024-09-27

**Authors:** Jozsef Nagy, Wolfgang Fenz, Veronika M. Miron, Stefan Thumfart, Julia Maier, Zoltan Major, Harald Stefanits, Johannes Oberndorfer, Nico Stroh, Vanessa Mazanec, Philip-Rudolf Rauch, Andreas Gruber, Matthias Gmeiner

**Affiliations:** 1 eulerian-solutions e.U., Leonfeldnerstraße 245, 4040 Linz, Austria; jozsef.nagy@eulerian-solutions.com; 2 Unit Medical Informatics, RISC Software GmbH, Softwarepark 32a, 4232 Hagenberg, Austria; wolfgang.fenz@risc-software.at (W.F.); stefan.thumfart@risc-software.at (S.T.); 3 Institute of Polymer Product Engineering, Johannes Kepler University Linz, Altenberger Strasse 69, 4040 Linz, Austria; veronika.miron@biz-up.at (V.M.M.); julia.maier@jku.at (J.M.); zoltan.major@jku.at (Z.M.); 4 Medical Faculty, Johannes Kepler University Linz, Altenberger Strasse 69, 4020 Linz, Austria; harald.stefanits@kepleruniklinikum.at (H.S.); johannes.oberndorfer@kepleruniklinikum.at (J.O.); nico.stroh@kepleruniklinikum.at (N.S.); vanessa.mazanec@kepleruniklinikum.at (V.M.); philip-rudolf.rauch@kepleruniklinikum.at (P.-R.R.); matthias.gmeiner@kepleruniklinikum.at (M.G.); 5 Department of Neurosurgery, Kepler University Hospital, Wagner-Jauregg-Weg 15, 4020 Linz, Austria; 6 Clinical Research Institute for Neuroscience, Johannes Kepler University Linz, 4020 Linz, Austria

**Keywords:** cerebral aneurysm, fluid–structure interaction simulation, hemodynamics, structural mechanics, initiation

## Abstract

**Background:** Hemodynamics during the growth process of cerebral aneurysms are incompletely understood. We developed a novel fluid–structure interaction analysis method for the identification of relevant scenarios of aneurysm onset. **Method:** This method integrates both fluid dynamics and structural mechanics, as well as their mutual interaction, for a comprehensive analysis. Patients with a single unruptured cerebral aneurysm were included. **Results:** Overall, three scenarios were identified. In scenario A, wall shear stress (WSS) was low, and the oscillatory shear index (OSI) was high in large areas within the region of aneurysm onset (RAO). In scenario B, the quantities indicated a reversed behavior, where WSS was high and OSI was low. In the last scenario C, a behavior in-between was found, with scenarios A and B coexisting simultaneously in the RAO. Structural mechanics demonstrated a similar but independent trend. Further, we analyzed the change in hemodynamics between the onset and a fully developed aneurysm. While scenarios A and C remained unchanged during aneurysm growth, 47% of aneurysms in scenario B changed into scenario A and 20% into scenario C. **Conclusions:** In conclusion, these findings suggest that WSS and the OSI are reciprocally regulated, and both low and high WSS/OSI conditions can lead to aneurysm onset.

## 1. Introduction

Cerebral aneurysms affect 2–5% of the general population [1]. An aneurysm rupture may cause a severe subarachnoid hemorrhage (SAH) with high mortality and morbidity rates [2]. Several studies have demonstrated various clinical, genetic, morphological, or hemodynamic factors contributing to the initiation, growth, and rupture of cerebral aneurysms [3].

Recent computational fluid dynamics (CFD) investigations revealed significantly different hemodynamics in ruptured versus unruptured aneurysms [4,5]. Hemodynamic phenomena can provide mechanical triggers, which are transduced into biological signals, leading to possible aneurysm growth and rupture [6].

Numerous attempts have been made to study the influence of the wall shear stress (WSS), one of the best-characterized fluid-dynamic parameters, on these processes [5,6,7,8]. Meng et al. described a novel view of aneurysm development, proposing that WSS plays a key mechanistic role, as suggested by their high-versus-low WSS theory [9]. In other studies, similar investigations were carried out, with conflicting results [10].

If the exact mechanisms of aneurysm formation could be determined, aneurysm rupture might be predicted and prevented [7]. Additionally, aneurysm growth may occur in up to 18% of affected patients and may be associated with aneurysm destabilization and rupture [2]. Similarly, other studies found a 12-fold increase in rupture risk in growing aneurysms [11].

Currently, several studies have analyzed hemodynamics in aneurysm initiation [7,8,12]. Although many detailed fluid dynamics simulation analyses, including complex flow patterns, were conducted [13,14,15,16,17,18,19,20,21,22], only a few studies have focused on structural dynamics calculations [23,24,25,26]. Most of these studies focused on abdominal aneurysms [24,25,26]. However, a comprehensive evaluation of individual hemodynamics should include the analysis of fluid as well as structural aspects using fluid–structure interaction (FSI) analysis. Therefore, basic research remains important for accurately characterizing and understanding mechanisms of cerebrovascular diseases to enable patient-specific treatment strategy optimization.

The aim of this study is to develop a novel FSI analysis method and investigate the impacts of both hemodynamics and structural mechanics on the initiation and growth of cerebral aneurysms in order to better understand the controversial dynamics of the initiation and growth process of cerebral aneurysms.

## 2. Methods

### 2.1. Patient Data

Patients with a single unruptured cerebral aneurysm treated at the Department of Neurosurgery, Kepler University Hospital Linz in 2020 were included for the identification of characteristic scenarios regarding the initiation and growth of cerebral aneurysms. Data were collected retrospectively, and fusiform and dissecting aneurysms were excluded. Additionally, we excluded large aneurysms with a size > 10 mm (n = 4) to minimize the effect of aneurysm size on the FSI analysis, as previously reported [7,27]. Overall, 44 patients were included (26 females, mean age 54 years; ranging from 34–76 years). Microsurgical or endovascular treatments were performed in 19 and 25 patients, respectively. The aneurysms were located at the internal carotid artery (n = 13), middle cerebral artery (MCA; n = 16), anterior communicating artery (n = 9), and posterior circulation (n = 6). This study was approved by the local ethics committee (Ethikkommission der medizinischen Fakultät der Johannes Kepler Universität; EK Nr: 1129/2022), and the requirement for the acquisition of informed consent from patients was waived, owing to the retrospective nature of the research.

### 2.2. Patient Image Data

We extracted the aneurysm geometries from medical image data obtained via digital subtraction angiography (DSA). Since these images showed only the cerebral blood vessels with high contrast and no other anatomical structures, segmentation was relatively straightforward and could be performed with intensity thresholding and minor editing of the voxel volume. A hydrodynamic entrance length of 5–10 times the inlet and outlet diameters was utilized for the inflow and outflow vessels, respectively, in order to define the cutoff positions during the reconstruction of the vascular geometry [28,29]. In case the quality of the DSA images did not allow such a length, the maximum possible length was taken. The segmentation was then converted into a surface mesh (STL format), the centerlines of the mesh were calculated, and inlet as well as outlet planes were placed perpendicular to these lines.

There are multiple alternatives to investigating the aneurysms onset. Possibly the most ideal approach is the investigation of de novo aneurysms, as shown with 10 aneurysms in [7]. Often, it is difficult to obtain patient data of a large number of de novo aneurysms; thus, in literature, a retrospective approach is used alternatively, where aneurysms are manually removed from the parent vessel. While this approach may have some limitations compared to the first approach, it is still mostly utilized in literature [8,27,30,31,32,33,34].

**Figure 1:**
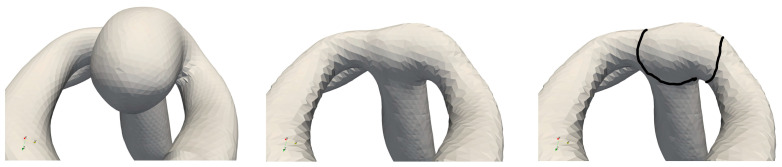
Typical CAD geometry of a cerebral artery, with aneurysm sac (**left**), aneurysm sac removed (**center**), and indicator line of the region of aneurysm onset (RAO, **right**).

In this work, the retrospective approach was utilized, due to the available patient images. The CAD models in the form of the generated STL files of the aneurysms (see Figure 1 left) were modified in a subsequent step by removing the aneurysm sac with the help of a Laplacian smoother algorithm [35,36,37,38] in order to obtain the geometry of the parent vessel without the aneurysm sac (see Figure 1 center).

In Figure 1, an estimated black line is shown, which indicates the approximate region of aneurysm onset (RAO). This region is important for understanding the initiation process of aneurysm growth.

The generated STL files (both with and without the aneurysm sac) were used to generate the volumetric calculation grid for the FSI simulations. In the first step, the fluid domain was established, and in the second subsequent step, the wall domain was created, with a constant wall thickness of 0.3 mm [22], by extruding and creating a separate vessel wall domain with the given thickness.

### 2.3. Hemodynamic and Structural Mechanical Modeling

A finite volume solver [39], based on the computational fluid dynamics framework OpenFOAM v2012 [40,41], alongside the fluid–structure interaction library solids4Foam [42], was utilized to numerically solve the unsteady equations of hemodynamics in the simulation process. The models applied the principles of mass and momentum conservation through both the continuity equation and the Navier–Stokes equations. For structural mechanics, Newton’s second law was employed. These equations were solved in a sequential manner and were coupled with a fluid–structure interaction (FSI) boundary condition. The details, as well as the experimental validation of the simulation methods, were described in [39].

The fluid dynamic boundary conditions at the inflow were defined by a pulsatile flow profile, characterized by a temporal velocity curve derived from published data [43]. The outflow conditions were set to a time-dependent value defined by experimental pressure measurements [44]. Boundary conditions along the interior vessel walls assumed a no-slip condition. Blood was represented in the model as a Newtonian fluid, with viscosity set at 0.04 poise and a constant density of 1.06 g/cm^3^. The simulation lasted for a single cardiac cycle of 1 s, corresponding to a heart rate of 60 beats per minute, discretized into 100 temporal increments (Δt = 0.01 s).

**Figure 2:**
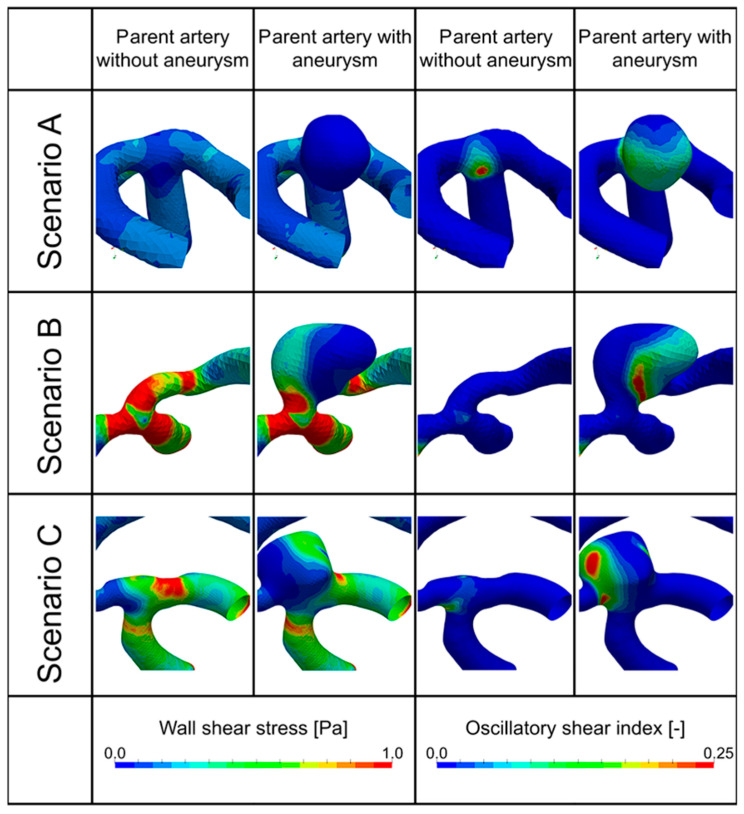
Hemodynamic scenarios. First two columns show the wall shear stress, the first column without the aneurysm sac and the second with the aneurysm sac; third and fourth columns show the oscillatory shear index OSI, the third column without the aneurysm sac and the fourth column with the aneurysm sac.

**Figure 3:**
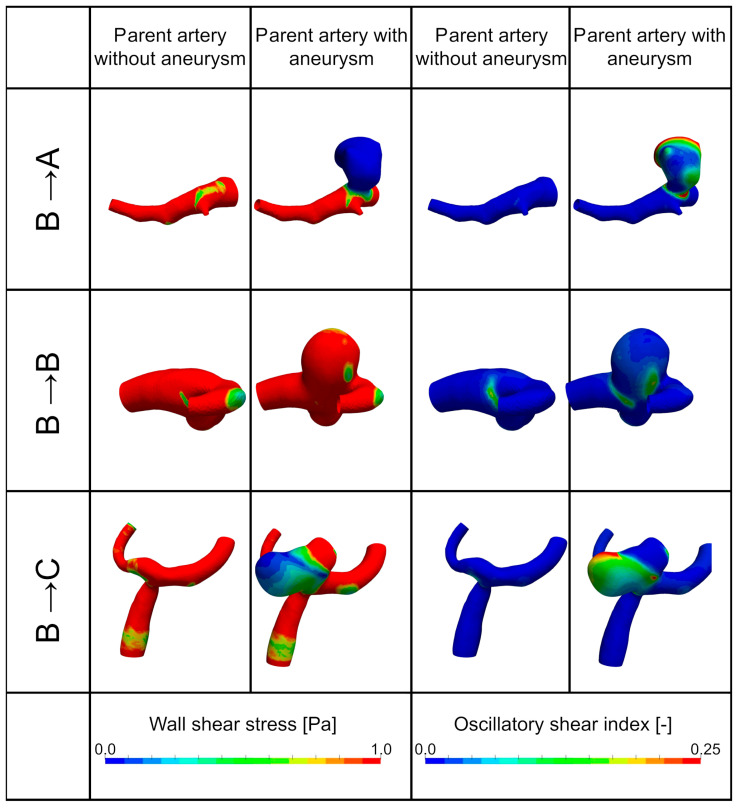
Change in hemodynamic features for scenario B. Individual rows show individual aneurysm geometries (row 1—scenario B to scenario A, row 2—scenario B remains scenario B, row 3—scenario B to scenario C); first two columns show the wall shear stress without the aneurysm sac (first column) and with the aneurysm sac (second column); third and fourth columns show the oscillatory shear index OSI without the aneurysm sac (third column) and with the aneurysm sac (fourth column).

**Figure 4:**
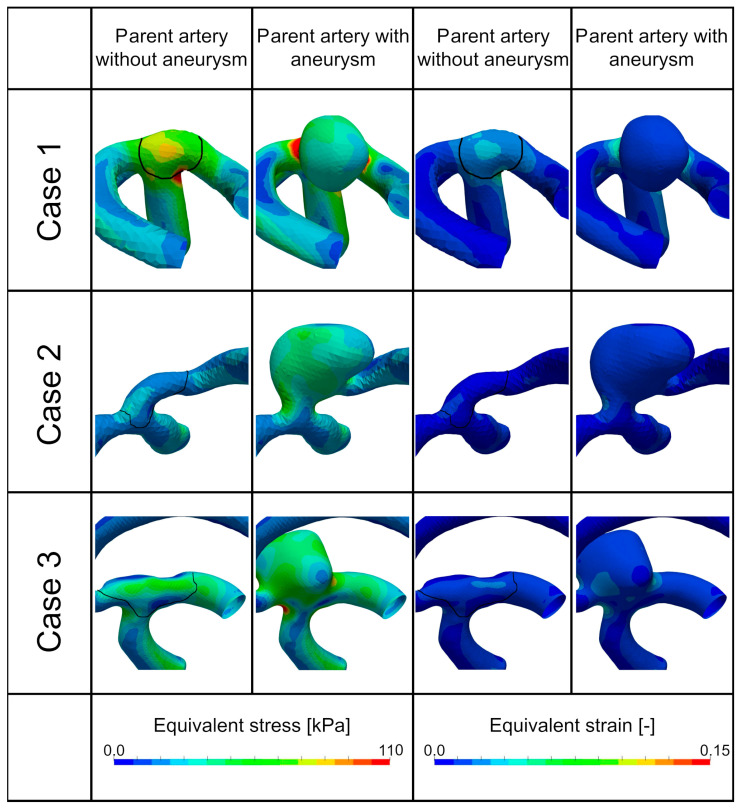
Structural dynamic features. The first two columns show the equivalent stress, first column without the aneurysm sac and the second column with the aneurysm sac; the third and fourth columns show the equivalent strain, the third column without the aneurysm sac and the fourth column with the aneurysm sac.

The vessel walls at the in- and outflow faces were fixed on the solid mechanical side. The vessel wall was assumed to be linear elastic, with a Young’s modulus value E of 2490 kPa [45] and a Poisson’s ratio ν of 0.49, which assumed an almost incompressible material, as described in [25,46]. Results for this Young’s modulus are shown in Figure 2, Figure 3 and Figure 4. We changed the Young’s modulus from the defined value of 2490 kPa from [45] to 5700 and 500 kPa [23], respectively, to assess the influence of vessel wall stiffness.

The investigated parameter of the oscillatory shear index OSI was defined as:

(1) OSI=0.51−∫0Tτdt∫0Tτdt,

where τ is the wall shear stress.

Investigated cohorts did not show normal distribution; thus, standard deviation was not given, and the significance of the difference between the means was determined with a Mann–Whitney U test (*p*-values < 0.05).

### 2.4. Convergence Analysis

In order to guarantee the accuracy of the simulations while maintaining a reasonable simulation runtime, the results of a convergence analysis are presented with the exemplary aneurysm in Figure 1. For this, the following numeric parameters were analyzed:

- Time step independence;
- Calculation mesh convergence;
- Newtonian vs. non-Newtonian fluid;
- One cardiac cycle vs. five cardiac cycles.

Table 1 shows the convergence results, including the required runtime for a simulation. A good time step convergence can be found, and a time step size of 0.01 s was utilized in all simulations, guaranteeing accuracy and also a reasonable simulation runtime.

The change in the fluid volume element edge length delivered a good convergence at 0.2 mm. The given values were target values for the meshing algorithm, and due to the irregularity of the geometry, small local deviations were possible.

The change in the solid volume element size was defined by the utilized number of cells over the thickness of the vessel wall; as in all simulations, a constant thickness of 0.3 mm [22] was utilized. Three cells over the thickness offered the best compromise between accuracy and simulation runtime.

The influence of a non-Newtonian viscosity model was also compared to the constant-viscosity approach by utilizing the Carreau–Yasuda model, as presented in [47]. Since the investigated aneurysms were located in arteries, the arterial blood flow with the assumed volume flux from [43] seemed to be dominated by high shear rates, thus resulting in a negligible change in fluid parameters, due to non-Newtonian effects, and no considerable change in structural mechanical parameters.

Since neither fluid nor solid mechanical properties were assumed to be viscoelastic, no time delay was introduced into the simulations. Due to the almost incompressibility of both the fluid as well as the vessel wall material [25,46], no significant differences can be seen when comparing one single cycle to five cycles.

Thus, all simulations were run with the base simulation settings of Δt = 0.01 s, Δx = 0.2 mm, 3 solid cells, constant viscosity, and one single cardiac cycle.

## 3. Results

### 3.1. Hemodynamic Scenarios in the Parent Artery and Cerebral Aneurysm

Three different scenarios can be identified (see first and third columns in Figure 2 for behavior without the aneurysm sac).

- In **Scenario A**, a large area with low WSS (<~0.1 Pa) and an increased OSI (>~0.1) was clearly visible.
- In **Scenario B**, high WSS values (i.e., large area with WSS > ~0.5 Pa) were observed, while OSI values remained low (<~0.05).
- In **Scenario C**, an intermediate behavior was observed, where certain parts of the region of aneurysm growth clearly showed low WSS, while other regions displayed high WSS values.

We did not find low WSS/low OSI or high WSS/high OSI scenarios in this study.

The results indicated that WSS and the OSI were reciprocally regulated. Table 2 shows the mean values of the three scenarios without an aneurysm sac. All three scenarios exhibited a significant difference (*p* < 0.05 in U-test), with scenario A clearly separating itself from the other two. A smaller yet still significant difference was found between scenarios B and C, with scenario C being in-between the two.

The change in Young’s modulus in the structural mechanical part of the simulations from 2490 to 5700 kPa did not affect the hemodynamic part considerably in the geometries without an aneurysm sac. Lowering the value to 500 kPa slightly modified it. Statistical differences between the scenarios remained significant.

In the geometries with aneurysm sacs (second and fourth column in Figure 2), a similar, reciprocally regulated behavior of WSS and the OSI was observed. Additionally, the WSS was lowered, compared to the geometries without an aneurysm sac, approximately by a factor of 5–15, due to the growth of the sac, with an increase in the OSI by a factor of approximately 2–3 in those regions. In Scenario C, the aneurysm sac could be clearly divided into two separate regions, where either WSS was high and the OSI value was low, or vice versa. Aneurysm growth was directed primarily in the direction of low WSS and high OSI in all cases with an aneurysm sac.

Table 3 shows the mean values of aneurysms belonging to the three scenarios for the geometries with an aneurysm sac. A significant difference between the scenarios was evident (all *p*-values < 0.05). Also, here, Scenario A showed a strong difference compared to scenario B, with scenario C being in-between the two.

The change in Young’s modulus from 2490 kPa to the higher value of 5700 kPa increased the average WSS in aneurysms and reduced the OSI. Introducing the lower value of 500 kPa lowered WSS and increased the OSI to a certain degree. Statistical differences between the scenarios remained significant.

Table 4 shows the distribution of scenarios in percentages amongst the patients with and without aneurysms. In geometries without aneurysms, scenarios B and C were more prominent with similar percentages, whereas scenario A was less likely. Strong contrast geometries with aneurysms showed a tendency towards scenario C. The percentage of scenario A was significantly increased, whereas scenario B dropped to approximately 11% of all cases. Despite the change in Young’s modulus, all scenarios remained unaltered.

Scenarios A and C did not change due to growth in any of the aneurysms. A very different behavior can be seen in geometries in scenario B. Although this scenario can be maintained in 33.42% of the cases, it can change to scenario A (46.65%), as well as to scenario C (19.93%). (Figure 3 shows illustrative cases).

### 3.2. Structural Mechanical Scenarios in the Parent Artery and Cerebral Aneurysm

Two scenarios (D and E) can be identified in the structural mechanical results; however, these scenarios did not fully correspond to the hemodynamic ones (A, B, and C). Thus, structural mechanics and hemodynamics were not directly correlated.

- **Scenario D** was characterized by regions with elevated equivalent wall stresses (MISES) (i.e., areas > ~70 kPa visible; see Figure 4, first row).
- **Scenario E** indicated regions with low equivalent wall stresses (i.e., areas mostly < ~50 kPa) in both the region of aneurysm onset and the aneurysm wall (see Figure 4, second row).

The aneurysm in the third row of Figure 4 showed a mixed behavior, where the parent vessel without a sac was characterized mostly by scenario E and the configuration with a sac by scenario D.

Equivalent strain values were relatively low (<0.1 [23]) in all geometries (except the simulation with low Young’s modulus; see model limitations for more details). Table 5 and Table 6 show the mean values of geometries without and with an aneurysm sac, respectively. The statistical difference in the conducted U-test was significant. There was increased stress, as well as strain, due to aneurysm growth.

The change in Young’s modulus (see Table 5 and Table 6) did not change the mean stress considerably, as due to the workflow in FSI, the force of the blood exerted by the blood pressure upon the vessel wall remained constant; however, the strain changed according to the linear material low in a mostly linear fashion.

The results suggested a certain dynamic between the two scenarios. In geometries without aneurysms, scenario E with lowered stress values was more prominent (see Table 7), whereas the initiation of an aneurysm clearly showed an increase in stress towards scenario D. Some Scenario E aneurysms (11.4%) remained in this scenario; however, most changed to scenario D. None of the geometries showed a decrease in stress after aneurysm growth (i.e., no change from scenario D towards E).

## 4. Discussion

### 4.1. Hemodynamics

The role of WSS in the aneurysm initiation process has recently been discussed, with divergent results [9]. Kulcsar and Meng suggested that the combination of high WSS and high positive spatial WSS gradient (WSSG) might play a role in aneurysm formation [9,12]. According to Fujimura, the aneurysmal initiation area may correspond to the highest WSS divergence (WSSD point) [7]. Zimny et al. analyzed hemodynamic changes in patients with MCA aneurysms and postulated that a higher WSS may impact aneurysm formation, whereas WSSG may promote this process [8].

In this study, using the distribution of WSS, as well as the OSI, the main hemodynamic processes of aneurysm initiation could be identified. Scenario B resembled patterns identified in previously published studies [9]. Scenario A clearly showed low WSS values in the RAO, along with high OSI values. Interestingly, in this work, additional aneurysms with a complex hemodynamic behavior were presented in-between, as illustrated in Scenario C; certain parts of the region of aneurysm growth clearly showed low WSS values, and other regions showed high WSS values. However, we did not observe a specific low WSS/low OSI or high WSS/high OSI scenario.

Aneurysm initiation has been considered to be partially dependent on chronic inflammation in the vascular wall [7]. Our results may indicate that these processes might be triggered by high WSS conditions, as well as high OSI conditions. However, WSS and the OSI seem to be reciprocally regulated in this stage of the disease. Similarly, for aneurysm growth, Meng et al. postulated the “high-versus-low wall shear stress” controversy, indicating the complexity of this disease. Our results seem to suggest that one of these alternatives in one aneurysm does not rule the other out in another one. While low WSS may be associated with an inflammatory cell-mediated pathway, high WSS may lead to a mural cell-mediated pathology. Both pathways may trigger aneurysm growth and rupture [9]. However, our results suggest that these two pathways may play a role in aneurysm initiation and can coexist.

The investigation revealed a significant dynamic between identified scenarios. WSS did not increase in any of the investigated geometries but decreased after aneurysm initiation. The only change occurred in scenario B, where WSS levels were either maintained (scenario B→B in Figure 3), lowered (scenario B→A in Figure 3), or partially lowered (scenario B→C in Figure 3). Again, the OSI was regulated in an inverse way. Aneurysms with low WSS and a high OSI (scenario A) did not change this behavior due to aneurysm initiation, and a mixed distribution of WSS and OSI (scenario C) was maintained after the start of growth. Our results provide evidence that the inflammatory cell-mediated pathway with low WSS and a high OSI might be the more dominant one during aneurysm initiation.

The analysis of vessel wall stiffness (three different Young’s moduli) strengthened our analysis. Changing the value from 2490 kPa [45] to the maximum value of 5700 kPa in [23] did not influence the hemodynamic behavior. Lowering the value to the lowest reported stiffness in [23] resulted in small changes; however, the identified behavior and dynamics of scenarios did not change.

### 4.2. Structural Mechanics

The structural mechanical behavior of the vessel and aneurysm wall appeared to be independent of the hemodynamics, showing two different scenarios (D and E). Although scenario D showed elevated stress values (>70–100 kPa), the stress level was still far away from the critical value of 421 kPa, as determined in [38], where rupture could be estimated. Similarly, in the other scenario with even lower stress values, rupture may not be expected.

Aneurysm initiation induced stress in the vessel wall in more than half of the investigated geometries. With the addition of cases with already elevated stress from the start, aneurysm initiation and growth clearly placed additional stress onto the vessel tissue rather than relieving it.

The ES in the geometries without and with an aneurysm sac were below 0.1. Cho et al. [23] provided a critical ES threshold value of 0.3, which was not reached in any of the geometries (with the exception of the simulations at a low Young’s modulus and with an aneurysm sac; see model limitations). In hemodynamics, while the decreased pathway of wall shear stress seemed to be the more dominant one, in the vessel wall, stresses and strains seemed to increase due to aneurysm growth. Since both stress and strain values were significantly below the critical thresholds, as identified in [23,38], respectively, increasing the structural mechanical quantities could be considered a compromise in favor of the preferable reduction in WSS and increase in the OSI in hemodynamics.

The influence of stiffness can be clearly seen in the change in the equivalent strain. By changing the Young’s modulus, the strain changed accordingly in an almost linear fashion. Stress values did not change, as they were defined by the FSI workflow via the force exerted by the blood pressure onto the vessel wall, which remained constant. The small change in strain for the geometries without an aneurysm sac resulted in a negligible or very small change in hemodynamics (see Table 2 and Table 5). The increased change in strain for the geometries with aneurysms resulted in a corresponding increased modification in hemodynamics (see Table 3 and Table 6). However, these changes could only be seen in the mean values, and the scenarios and their dynamics still remained unaltered.

Both the behaviors in stress and strain underline the fact that none of the aneurysms ruptured in reality. However, our results suggest that aneurysm initiation and growth are mainly associated with a reduction in WSS, along with an increase in stress in the vessel wall.

### 4.3. Model Limitations

Our investigation had certain limitations due to uncertainties in input data, which had to be approximated with certain assumptions. The assumption was made that during aneurysm development, the main shape of the artery does not change considerably (e.g., angle of outflow arteries, etc.). In the FSI simulations, an assumption regarding fixing the geometry must be made. Here, the blood vessel was fixed at the ends of the inflow vessel, as well as the outflow vessels. This does not correspond to real life, as the blood vessels are connected to other vessels. Additionally, the investigated parts may be in contact with parts of brain tissue as well. This contact was neglected in the simulations.

Parent vessels without aneurysms were created by manually removing the sac. Thus, this manual removal introduced a degree of subjective error. Therefore, extra attention was paid during aneurysm removal to minimize such subjective user error.

For the generation of the investigated scenarios, only unruptured but treated aneurysms were considered to avoid the influence of the change in shape due to rupture. In the next step, the influence of ruptured aneurysms will be investigated.

In this work, a constant vessel wall thickness of 0.3 mm was assumed [22]. This topic is currently under investigation, and further studies on wall thickness are needed in future steps to advance toward a more reliable description of aneurysm behavior.

Simulations with an aneurysm sac with the lowest reported value of Young’s modulus in [23] showed increased strain values of >0.5, where the limits of the utilized linear elastic model were reached. This stiffness was considered to be a worst-case scenario for blood vessels. In future investigations, a non-linear approach will be utilized to better describe the material behavior at high strain values.

The data for the pulsatile blood flow was taken from experimental data found in the literature [36]. In experiments, systematic errors occur, which add additional uncertainty into the simulations. Since there was no information on the material properties for the tissues of the individual investigated aneurysms in this work, an assumption from literature was made for the Young’s modulus of the material.

## 5. Conclusions

Our simulations suggest that hemodynamics, through the reciprocal regulation of WSS and OSI, are closely associated with aneurysm initiation and growth. Hemodynamics and structural mechanics appear to present separate and independent scenarios, with two hemodynamic scenarios remaining stable during growth, while the third showed a tendency towards change. Structural mechanics indicate increases in stress and strain as a result of aneurysm growth. It is important to note that our analysis was limited to unruptured aneurysms, which presents a limitation in fully understanding the rupture risk. Further, although 44 patients were carefully analyzed, we need to confirm these results in a prospective multi-center investigation. However, we believe our findings provide a solid foundation for future research aimed at predicting aneurysm rupture risk by considering their morphology, hemodynamics, and structural mechanics.

## Figures and Tables

**Table 1:** Convergence analysis of time step size and mesh size independence, as well as the influence of the non-Newtonian viscosity model and multiple cardiac cycles. (Base simulation settings consisted of Δt = 0.01 s, Δx = 0.2 mm, 3 solid cells, Newtonian model, and one single cardiac cycle).

	Parameter	WSS [Pa]	OSI [-]	ES [-]	MISES [kPa]	Runtime [s]
Time independence	Δt = 0.1 s	0.083	0.148	0.033	73.54	347
	Δt = 0.01 s	0.085	0.150	0.034	75.41	715
	Δt = 0.001 s	0.086	0.151	0.035	75.43	1872
Fluid mesh convergence	Δx = 0.3 mm	0.080	0.139	0.031	69.84	262
	Δx = 0.25 mm	0.083	0.145	0.033	72.14	426
	Δx = 0.2 mm	0.086	0.150	0.034	75.41	715
	Δx = 0.15 mm	0.088	0.152	0.035	76.11	1592
Solid mesh convergence	2 cells	0.086	0.150	0.032	73.94	578
	3 cells	0.086	0.150	0.034	75.41	715
	4 cells	0.086	0.151	0.034	75.42	1643
Viscosity model	Newtonian	0.085	0.150	0.034	75.41	715
	Non-Newtonian	0.086	0.151	0.034	75.41	727
Number of cardiac cycles	1	0.085	0.150	0.034	75.41	715
	5	0.085	0.150	0.034	75.41	3512

**Table 2:** Mean values of scenarios A, B, and C, as well as *p*-values between scenarios without aneurysms.

Young’s Modulus		Mean Values			*p*-Values		
		A	B	C	A vs. B	A vs. C	B vs. C
2490 kPa	WSS [Pa]	0.261	1.772	1.176	1.90 × 10^−4^	8.86 × 10^−4^	3.82 × 10^−2^
	OSI [-]	0.075	0.018	0.03	7.52 × 10^−4^	2.88 × 10^−3^	4.22 × 10^−2^
5700 kPa	WSS [Pa]	0.259	1.768	1.164	1.86 × 10^−4^	8.74 × 10^−4^	3.73 × 10^−2^
	OSI [-]	0.077	0.020	0.032	7.59 × 10^−4^	2.92 × 10^−3^	4.29 × 10^−2^
500 kPa	WSS [Pa]	0.231	1.568	1.040	1.88 × 10^−4^	8.81 × 10^−4^	3.83 × 10^−2^
	OSI [-]	0.084	0.020	0.034	7.56 × 10^−4^	2.89 × 10^−3^	4.25 × 10^−2^

**Table 3:** Mean values of scenarios A, B, and C, as well as *p*-values between scenarios with aneurysms.

Young’s Modulus		Mean Values			*p*-Values		
		A	B	C	A vs. B	A vs. C	B vs. C
2490 kPa	WSS [Pa]	0.025	0.39	0.193	3.00 × 10^−3^	8.54 × 10^−4^	1.83 × 10^−2^
	OSI [-]	0.154	0.037	0.056	1.23 × 10^−2^	3.72 × 10^−3^	3.22 × 10^−2^
5700 kPa	WSS [Pa]	0.034	0.441	0.243	1.98 × 10^−3^	6.37 × 10^−4^	1.13 × 10^−2^
	OSI [-]	0.113	0.033	0.044	9.54 × 10^−3^	2.27 × 10^−3^	1.92 × 10^−2^
500 kPa	WSS [Pa]	0.016	0.286	0.137	2.87 × 10^−3^	7.23 × 10^−4^	1.95 × 10^−2^
	OSI [-]	0.241	0.050	0.078	1.33 × 10^−2^	3.89 × 10^−3^	3.12 ×10^−2^

**Table 4:** Percentage of hemodynamic scenarios with and without aneurysms.

	A	B	C
without aneurysm	22.7%	34.1%	43.2%
with aneurysm	38.6%	11.4%	50.0%

**Table 5:** Mean values of scenarios D and E, as well as *p*-values between scenarios without aneurysms.

Young’s Modulus		Mean Values		*p*-Value
		D	E	D vs. E
2490 kPa	MISES [kPa]	76.5	45.8	1.27 × 10^−3^
	ES [-]	0.033	0.019	4.14 × 10^−3^
5700 kPa	MISES [kPa]	77.3	46.3	1.65 × 10^−3^
	ES [-]	0.014	0.008	4.89 × 10^−3^
500 kPa	MISES [kPa]	77.0	46.2	1.33 × 10^−3^
	ES [-]	0.167	0.095	4.43 × 10^−3^

**Table 6:** Mean values of scenarios D and E, as well as *p*-values between scenarios with aneurysms.

Young’s Modulus		Mean Values		*p*-Value
		D	E	D vs. E
2490 kPa	MISES [kPa]	83.6	49.0	1.55 × 10^−4^
	ES [-]	0.103	0.046	1.38 × 10^−3^
5700 kPa	MISES [kPa]	84.2	49.3	2.67 × 10^−4^
	ES [-]	0.043	0.019	3.39 × 10^−3^
500 kPa	MISES [kPa]	83.9	49.1	1.94 × 10^−4^
	ES [-]	0.515	0.23	1.77 × 10^−3^

**Table 7:** Percentage of structural mechanic scenarios with and without aneurysms.

	D	E
without aneurysm	43.2%	56.8%
with aneurysm	88.6%	11.4%

## Data Availability

The data are not publicly available due to the fact that authors want to keep patient data private, however data can be made available upon request.
